# Epidemiology of neonatal infections in hospitals of Nepal: evidence from a large- scale study

**DOI:** 10.1186/s13690-020-00424-z

**Published:** 2020-05-07

**Authors:** Shyam Sundar Budhathoki, Avinash K. Sunny, Pragya Gautam Paudel, Jeevan Thapa, Lila Bahadur Basnet, Sandeepa Karki, Rejina Gurung, Prajwal Paudel, Ashish KC

**Affiliations:** 1 Golden Community, Lalitpur, Nepal; 2 Department of Primary Care and Public Health, School of Public Health, Imperial College London, London, UK; 3 Department of Community Health Sciences, Patan Academy of Health Sciences, Lalitpur, Nepal; 4 School of Public Health & Community Medicine, B P Koirala Institute of Health Sciences, Dharan, Nepal; 5 Ministry of Health and Population, Government of Nepal, Ramshah Path, Kathmandu, Nepal; 6 Department of Women’s and Children’s Health, Uppsala University, Dag Hammarskjölds väg 14B, Uppsala, Sweden

**Keywords:** In-patient neonatal infection, Antenatal check-up, Caesarean section, Birth asphyxia, Risk factor, Nepal

## Abstract

**Background:**

Every year, neonatal infections account for approximately 750,000 neonatal deaths globally. It is the third major cause of neonatal death, globally and in Nepal. There is a paucity of data on clinical aetiology and outcomes of neonatal infection in Nepal. This paper aims to assess the incidence and risk factors of neonatal infection in babies born in public hospitals of Nepal.

**Methods:**

This is a prospective cohort study conducted for a period of 14 months, nested within a large-scale cluster randomized control trial which evaluated the Helping Babies Breathe Quality Improvement package in 12 public hospitals in Nepal. All the mothers who consented to participate within the study and delivered in these hospitals were included in the analysis. All neonates admitted into the sick newborn care unit weighing > 1500 g or/and 32 weeks or more gestation with clinical signs of infection or positive septic screening were taken as cases and those that did not have an infection were the comparison group. Bivariate and multi-variate analysis of socio-demographic, maternal, obstetric and neonatal characteristics of case and comparison group were conducted to assess risk factors associated with neonatal infection.

**Results:**

The overall incidence of neonatal infection was 7.3 per 1000 live births. Babies who were born to first time mothers were at 64% higher risk of having infection (aOR-1.64, 95% CI, 1.30–2.06, *p*-value< 0.001). Babies born to mothers who had no antenatal check-up had more than three-fold risk of infection (aOR-3.45, 95% CI, 1.82–6.56, *p*-value< 0.001). Babies born through caesarean section had more than two-fold risk (aOR-2.06, 95% CI, 1.48–2.87, *p*-value< 0.001) and babies with birth asphyxia had more than three-fold risk for infection (aOR-3.51, 95% CI, 1.71–7.20, p-value = 0.001).

**Conclusion:**

Antepartum factors, such as antenatal care attendance, and intrapartum factors such as mode of delivery and birth asphyxia, were risk factors for neonatal infections. These findings highlight the importance of ANC visits and the need for proper care during resuscitation in babies with birth asphyxia.

## Background

In 2018 the global neonatal death rate was 18 per 1000 live births, accounting for 2.5 million neonatal deaths [1]. This represents approximately 7000 neonatal deaths every day [1]. Among under five mortalities, more than two-fifth of deaths occur during the neonatal period and one third of these neonatal deaths are due to infection [2]. Among these deaths, 25% occur in South Asia and sub-Saharan Africa [2].

Neonatal infection is manifested by systemic signs of infection and isolation of a bacterial or other pathogen from the bloodstream [3, 4]. Infection in newborn increases the risk of developing neurodevelopmental impairments such as delayed gross motor, language and cognitive skills later in pre-school life [5–7]. Compared to high income countries, low-and middle-income countries (LMICs) have approximately 40 times higher incidence rates of neonatal infection and double the mortality rates [2, 3]. However, there is limited population-based evidence available from low-income countries and a lack of standardised diagnostic criteria and definitions which together serve as obstacles to accurate estimation of the global burden of neonatal infections [3].

Nepal aims to reduce neonatal mortality rate to 12 per 1000 live birth or less by 2030 as part of the target set by the Sustainable Development Goal for health [8, 9]. Between 2001 to 2016, the neonatal mortality rate in Nepal declined from 39 to 21 deaths per thousand [10]. However, Neonatal infection is one of the leading causes of hospital admissions and neonatal deaths in Nepal [11–13]. The prevalence of neonatal infections ranges between 2 and 4% in Nepal [12], with 37.1% of infections occurring in neonatal intensive care units of tertiary referral hospital [11]. Previous study on exploring trends and determinants of neonatal mortality in Nepal identified several risk factors of neonatal mortality in Nepal [10]. These risk factors include lack of education, less than four antenatal care visits, babies born with less than two-year birth intervals and mother’s exposure to indoor air pollution [10].

In Nepal, maternal and newborn health service is delivered through three tier approach [14, 15]. Primary health care center provides routine antenatal care service, normal vaginal delivery and basic newborn care. District or secondary level hospital provide basic emergency obstetric services and in-patient management of sick newborns. Regional or tertiary level hospital provide comprehensive emergency obstetric services and specialized newborn care to sick newborns.

Early identification of risk factors of neonatal infections would help to select babies who need special care. This study aims to add up to the evidence of incidence and risk factors of neonatal infection in district and regional hospitals of Nepal.

## Methods

### Study setting, design and period

**Fig. 1:**
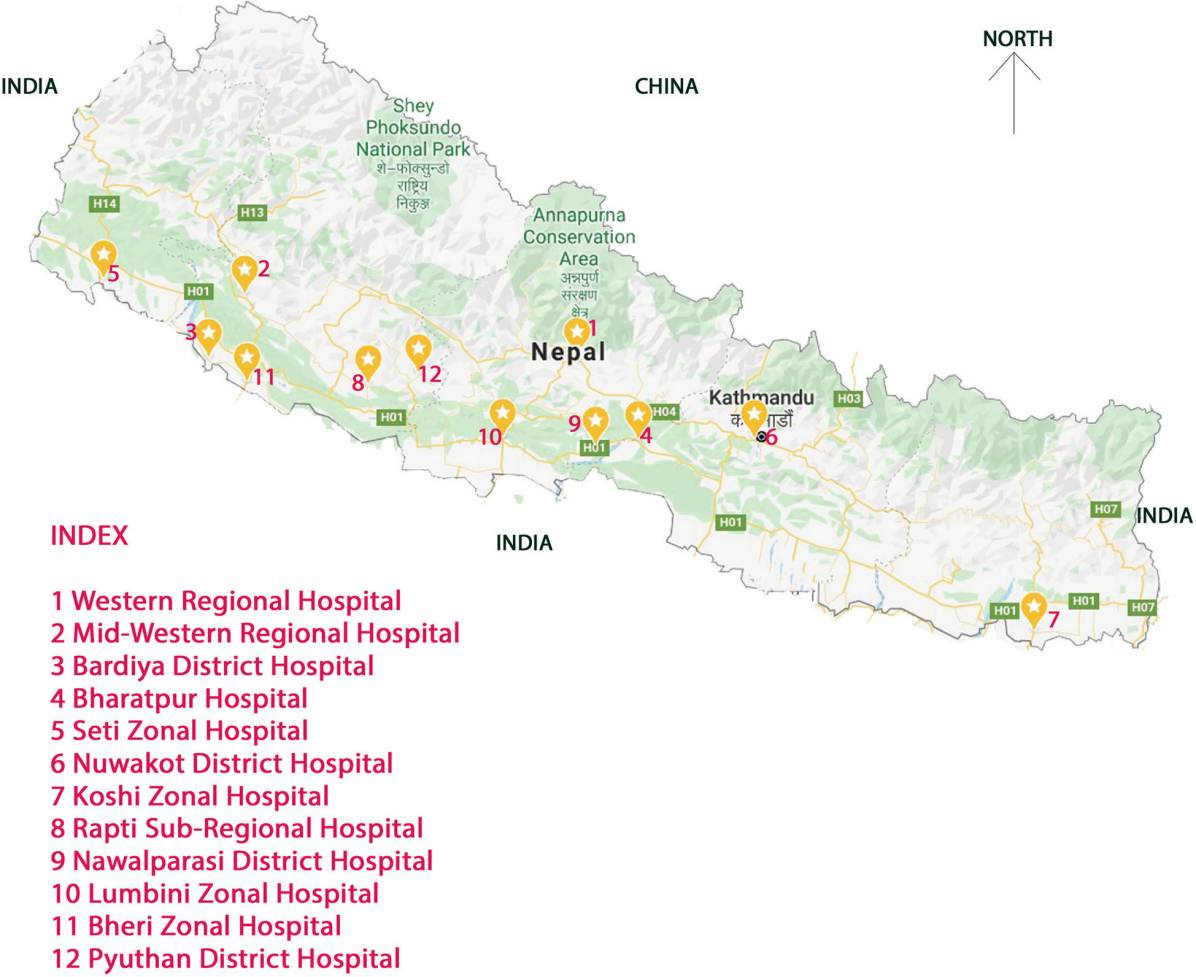
Location of hospitals pinned within the Map of Nepal

This is a prospective cohort study, nested within a large-scale cluster randomized control trial which evaluated the Helping Babies Breathe Quality Improvement package in 12 public hospitals of Nepal [16, 17]. The hospitals are mapped in Fig. 1. These hospitals are referral centres which provide obstetric, neonatal and paediatric services. The annual number of deliveries in each hospital ranged from 1194 to 11,318. All these hospitals provided comprehensive obstetric and neonatal care services along with sick newborn care services (Additional file 1). This paper presents data from a period of 14 months from July 2017 to August 2018.

#### Study population

All the newborns who were born at the participating hospitals during the study period were eligible for the study. The newborns with consenting parents were enrolled in the study. All the newborns with signs of clinical infection or positive septic screening with birth weight 1500 g or more and/or gestational age 32 weeks or more were considered as ‘cases’. All other newborns were considered as referent or comparison population. The cases were treated using antibiotics was per the national guideline [12].

#### Study size

All eligible cases consenting for participation were included in the study. A total of 60,400 mothers, based on the sample size required to evaluate the effect of quality improvement package for the primary study was taken for this study.

#### Data sources/measurement

Information on newborn were obtained from data collectors who were assigned to the maternity ward in each hospital. A data retrieval form was used to extract clinical information on mothers and newborn from the patient records and register. A semi-structured interview with mothers were conducted to assess information on their socio-demographic characteristics and antenatal care.

#### Data management and statistical methods

After the completion of recording and interviews, forms were assessed by the data coordinator in each site for completeness. To ensure the accuracy of the data collected, 10% of the mothers’ information were recollected by the data coordinator. At the end of each day, the information sheets were indexed by the data coordinator. Every week completed forms were sealed in an opaque envelope and sent to the Kathmandu office for further data management. In the Kathmandu office, these forms were reassessed for completeness and open-ended questions were recoded. Data entry was done in CS pro (Census and Survey Processing System) database and 5 % of data were re-entered to assess the accuracy of data entry. Every month the data were entered into a data entry platform, CS pro. Finally, the data were exported to SPSS (Statistical Package for the Social Sciences) for statistical analysis.

To ensure the privacy and safety of the data, the exported data were stored in an external hard drive. Prior to data analysis, anonymization and removal of location of the participants was ensured. All hard copies of information sheets were indexed and stored as per the ethical guideline.

### Study variables

Socio-demographic, maternal, obstetric and neonatal characteristics were collected through data extraction and semi-structured interviews.

#### Maternal age

Maternal age was categorized as less than 20 years, 20–35 years and 35 years and above.

#### Maternal education

Mothers who are illiterate or have received education through informal trainings other than in schools were categorised as having ‘no formal education’ while those who had gone to school for education were considered as having ‘formal education’.

#### Ethnicity

was categorized as Dalit, Janjati, Madhesi, Muslim, Chettri/Brahmin, and other castes based on hierarchical caste system of Nepal [18]. Ethnicity was categorized as disadvantageous group (Dalit, Janjati and Muslim) and relatively advantageous group (Madhesi, Chettri/Brahmin and other castes).

#### Mothers smoking status

Smokers were those who had a history of smoking during or before pregnancy. Non-smokers were those who never smoked in their lifetime.

#### Indoor tobacco smoke

Environmental tobacco smoke (ETS), also referred to as second hand smoke, is a mixture of exhaled mainstream smoke (MS) and side stream smoke (SS) released from the smouldering tobacco product [19],

#### Parity

Mothers who had no previous births (nulliparity), at least one or more previous birth (primiparity and multiparity),

#### Antenatal check-up

Mothers who received antenatal care (ANC) check-up from a skilled provider,

#### Four antenatal check-up

Mothers who received at least four ANC check-ups from a skilled provider or less than four check-ups,

#### Severe anaemia during pregnancy

Serum haemoglobin less than 7.0 g/decilitre,

#### Suspected maternal infections

Mothers who received prophylactic antibiotics for a suspected infection,

#### Mode of delivery

Mothers who gave birth vaginally or through caesarean section.

#### Gender of the baby

The sex of the baby as male or female.

#### Weight of the baby

Birth weight categorized as less than 2500 g, 2500–4000 g or 4000 g and more.

#### Gestational age

Gestational age is calculated using the last menstrual period and categorized as less than 37 weeks, 37–42 weeks or 42 weeks and more**.**

#### Immediate breast feeding

Breast feeding within 1 h of birth.

#### Applied antiseptic to umbilical cord stump

Application of antiseptic to the umbilical cord**.**

#### Multiple birth

Mother delivered two or more babies.

#### Birth asphyxia

Birth asphyxia was defined as APGAR score of less than 6 at 1 min or/and APGAR score of less than 6 at 5 min.

### Data analysis

The incidence of neonatal infection was calculated with 95% confidence interval (CI) by socio-demographic characteristics (maternal age, maternal education, ethnicity, smoking and indoor pollution), maternal characteristics (parity, antenatal checkup and severe anemia), obstetric and neonatal characteristics (suspected maternal infection, mode of delivery, gender of baby, birth weight, gestational age, breast feeding, applied antiseptic to umbilical cord, multiple birth and birth asphyxia). The variables among the socio-demographic, maternal, obstetric and neonatal characteristics for neonatal infection with 95% CI higher than the comparator group with *p* < 0.05 were included within bi-variate logistic regression. Variables included within the bi-variate analysis with a *p*-value < 0.01 were subsequently included within the multi-variate analysis. Crude odds ratios were calculated from bi-variate analysis and adjusted odds ratio were calculated from multi-variate analysis.

Missing variables were excluded from the analysis.

## Results

**Fig. 2:**
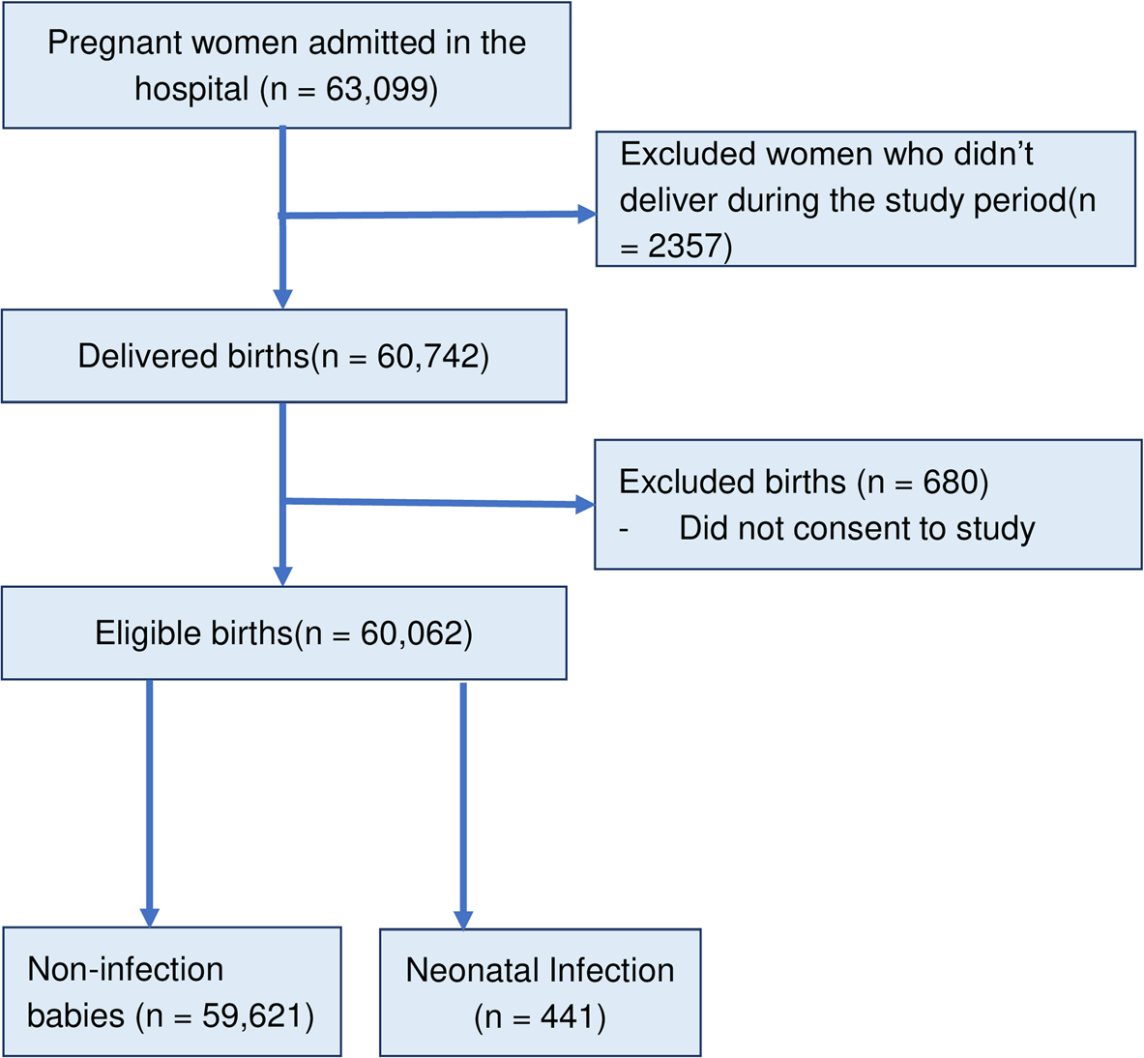
Study flow diagram

A total of 63,099 pregnant women were admitted to the selected hospitals with 60,742 having delivered during the study period. Of those, 60,062 babies were eligible for the study and 441 of them were admitted as Neonatal infection (Fig. 2).

Incidence of Neonatal infection was high among babies of advantageous ethnic group (0.9, 95% CI, 0.8–1.0) in comparison with babies of disadvantageous ethnic group (0.6, 95% CI, 0.6–0.7). The incidence was higher among babies born from mothers who had given birth for the first time (0.9, 95% CI, 0.8–1.0) than among babies of mothers who had given birth previously (0.6, 95% CI, 0.5–0.6). The incidence was also higher among babies of mothers who did not receive any antenatal check-ups by skilled providers (2.1, 95% CI, 1.2–3.7) than babies of mothers who received antenatal check-up (0.7, 95% CI, 0.6–0.7) (Table 1).

**Table 1:** Incidence of neonatal infection by socio-demographic and maternal characteristics

Socio-demographic and maternal characteristics	Neonatal infection		Incidence (95% CI)	*p*-value
Maternal age	No	Yes		
Less than 20 years	8346	57	0.7% (0.5–0.9)	0.621
20–35 years	50,176	370	0.7% (0.7–0.8)	
More than 35 years	1540	14	0.9% (0.5–1.5)	
Maternal education^a^				
No formal education	7863	61	0.8% (0.6–1.0)	0.309
Formal education	42,521	286	0.7% (0.6–0.7)	
Ethnicity				
Advantageous group	23,626	205	0.9% (0.8–1.0)	0.002
Relatively disadvantageous group	36,436	236	0.6% (0.6–0.7)	
Smoking/Indoor tobacco smoke				
Maternal smoking^b^	5679	26	0.5% (0.3–0.7)	0.026
Indoor tobacco smoke^a^	12,947	61	0.5% (0.4–0.6)	0.026
Parity				
0 previous birth	29,564	267	0.9% (0.8–1.0)	< 0.001
1 or more previous birth	30,498	174	0.6% (0.5–0.6)	
Antenatal check-up^a^				
No ANC	526	11	2.1% (1.2–3.7)	< 0.001
At least once	49,858	336	0.7% (0.6–0.7)	
Four antenatal check up				
Less than 4 ANC	11,542	61	0.5% (0.4–0.7)	0.029
4 or more ANC	38,316	275	0.7% (0.6–0.8)	
Severe maternal anemia	167	1	0.5% (0.1–3.5)	0.562

^a^missing-9678, ^b^missing-9680

The incidence of neonatal infection was higher among mothers who had suspected infection (1.3, 95% CI, 1.1–1.5) than those who did not have suspected infection (0.6, 95% CI, 0.6–0.7). Babies who were born through caesarean section (CS) showed a higher incidence of infection (1.3, 95% CI, 1.1–1.5) than babies born via vaginal delivery (0.6, 95% CI, 0.5–0.7). The incidence of neonatal infection was also higher among babies who had birth asphyxia (2.7, 95% CI, 1.5–4.7) than those who did not have birth asphyxia (Table 2).

**Table 2:** Incidence of neonatal infection by obstetric and neonatal characteristics

Obstetric and birth characteristics	Neonatal infection		Incidence (95% CI)	*p*-value
	No	Yes		
Suspected maternal infection^a^				
Yes	9893	128	1.3% (1.1–1.5)	< 0.001
No	40,993	263	0.6% (0.6–0.7)	
Mode of delivery				
Vaginal	47,059	276	0.6 (0.5–0.7)	< 0.001
Caesarean section	12,492	160	1.3 (1.1–1.5)	
Gender				
Boys	32,401	263	0.8% (0.7–0.9)	0.016
Girls	27,661	178	0.6% (0.5–0.7)	
Birth weight				
Less than 2500 g	6659	57	0.8% (0.6–1.0)	0.011
2500-4000 g	52,067	366	0.7% (0.6–0.8)	
4000 g or more	1336	18	1.3% (0.8–2.1)	
Gestational age				
Less than 37 weeks	46	5569	0.8% (0.6–1.0)	0.400
37 weeks or more	395	54,493	0.7% (0.7–0.8)	
Early breast feeding	43,292	301	0.7% (0.5–0.9)	0.903
Applied antiseptic to umbilical cord	18,547	126	0.7% (0.6–0.8)	0.395
Multiple birth				
No	59,698	437	0.7% (0.7–0.8)	0.414
Yes	364	4	1.1% (0.4–2.9)	
Birth asphyxia	413	11	2.7% (1.5–4.7)	< 0.001

^a^missing-9176

Bivariate regression analysis showed that babies who were born to mothers within advantageous ethnic group had a 34% higher risk of having an infection than babies born to mothers from disadvantageous ethnic group (cOR-1.34, 1.11–1.62, *p*-value = 0.002). Babies who were born to first-time mothers had a 59% higher risk of infection compared to primiparous or multiparous mothers (cOR-1.59, 95% CI, 1.31–1.92, *p*-value< 0.001). Babies who were born through CS had more than two-fold risk of infection than those born via vaginal delivery (cOR-2.20, 95% CI, 1.81–2.68, *p*-value< 0.001). Babies who had birth asphyxia had more than three-fold risk of infection than those who did not have birth asphyxia (cOR-3,77, 95% CI, 2.05–6.91, *p*-value< 0.001) (Table 3).

**Table 3:** Multi-variate and bi-variate analysis of the risk factor

Variables	crude Odds Ratio (cOR)	*P*-value	adjusted Odds Ratio (aOR)	*P*-value
Advantageous group (ref- disadvantageous group)	1.34 (1.11–1.62)	0.002	1.28 (1.03–1.60)	0.028
0 previous birth (ref- ≥1 previous birth)	1.59 (1.31–1.92)	< 0.001	1.64 (1.30–2.06)	< 0.001
No Antenatal check up (ref- with Antenatal check up)	3.15 (1.72–5.78)	< 0.001	3.45 (1.82–6.56)	< 0.001
Suspected maternal infection (ref- no maternal infection)	2.03 (1.64–2.51)	< 0.001	1.13 (0.80–1.59)	< 0.001
Caesarean section (ref- vaginal delivery)	2.20 (1.81–2.68)	< 0.001	2.06 (1.48–2.87)	< 0.001
Birth asphyxia (ref- no birth asphyxia)	3.77 (2.05–6.91)	< 0.001	3.51 (1.71–7.20)	0.001

In the multi-variate analysis, babies born to mothers who had no antenatal check-up had more than three-fold risk of neonatal infection (aOR-3.45, 95% CI, 1.82–6.56, p-value< 0.001). Babies born through CS had more than two-fold risk for infection (aOR-2.06, 95% CI, 1.48–2.87, p-value< 0.001) and babies who had birth asphyxia had more than three-fold risk for infection (aOR-3.51, 95% CI, 1.71–7.20, p-value = 0.001) (Table 3).

## Discussion

Our finding suggesting that babies born to first time mothers were at high risk of neonatal infection is consistent with the study conducted in Nepal by Shah et al. which reported that there was high risk of neonatal infection in babies born to nulliparous mothers [20]. Similar findings were observed in another study conducted by Adatara et al. [21]. This may be related to pregnancy complications commonly present in young mothers [22]. It is possible that young mothers lack experiences in handling babies, so might have a poor hygienic practice during postnatal period, which could lead to infection [23].

Babies born through CS had significantly higher risk of having infection than those born through vaginal delivery. This finding is consistent with the study conducted by Rojas et al. which demonstrated a significant association of CS with neonatal infection [24]. The intra-operative procedure during CS with a lack of fully sterile conditions might have led to the increased risk of infection [25]. Furthermore, the indications for CS such as fetal distress, cord prolapse, prolonged or obstructed labor contributes to the increased risk for infection [26, 27].

The findings in this study showed that babies with birth asphyxia had two-fold higher risk of neonatal infection. This is consistent with a study conducted by Getabelew et al. which demonstrated a three-fold increased risk of infection among babies who had birth asphyxia compared with those who did not [28]. Hospital acquired infections may occur when babies born with asphyxia are transferred to sick newborn care units for further management where the units are not in a sterile condition [29].

We also observed that babies of mothers who had no antenatal checkup by skilled providers had high risk of neonatal infection. This may be due to lack of counseling from the skilled providers for delivery preparations.

Our findings also showed that babies born to advantageous ethnic group had a higher risk of infection than those born to disadvantageous ethnic groups. In Nepal, the social and cultural practices relating to pregnancy care vary by ethnic group. Pregnant women from advantageous ethnic group have more restrictions in the hygiene practice than disadvantageous group, which might have led to risk for neonatal infection. Many cultural practices of advantageous groups like wrapping newborn with old clothes, applying oil to umbilical cord and poor breast feeding practices can result in neonatal infection. A study in a rural Nepal showed that the level of awareness among disadvantageous group is better than advantageous ethnic group [30].

### Methodological consideration

There are some limitations in the study. The study did not analyse some of the risk factors associated with neonatal infections such as previous medical history and previous preterm births. Furthermore, the interviews conducted with the mothers may be exposed to recall bias. This study has many strengths, including that it is a sample of over 60,000 birth from 12 different hospitals. Therefore, the results of this study are likely to be representative of the hospital based incidence rate of neonatal infection in Nepal.

## Conclusion

This study utilized data from a large cohort of births registered in public hospitals across Nepal to provide a better understanding of the neonatal infection from an epidemiological perspective. Findings from this study suggest that first time mothers, non-attendance at antenatal check-ups, CS and birth asphyxia are factors which increase the risk for neonatal infection. These findings suggest that there is a need to improve the intra-operative and post-operative environment to prevent infection of babies and mothers. Furthermore, improving care during the intrapartum period may reduce the risk for infection. Further research on what practices during the pregnancy in the advantageous ethnic group is needed to understand the link between neonatal infection and ethnicity in Nepal.

## Supplementary information

**Additional file 1.** Estimated deliveries at the selected hospital 2015.

## Data Availability

The data will be made available on request.

## Abbreviations

LMICs: Low- and middle-income countries
ANC: Antenatal care
CSPro: Census and survey processing system
SPSS: Statistical package for the social sciences
aOR: Adjusted odds ratio
