# Supplementary material for: Epidemiology of neonatal infections in hospitals of Nepal: evidence from a large- scale study
Source: Arch Public Health. 2020 May 7;78:39. doi: 10.1186/s13690-020-00424-z (PMC7203977; doi:10.1186/s13690-020-00424-z)
Supplement: Supplementary file 1 — Additional file 1. Estimated deliveries at the selected hospital 2015. [file 13690_2020_424_MOESM1_ESM.docx]

Additional file 1. Estimated deliveries at the selected hospital 2015

| **Name of hospital** | **Total deliveries per year (2015)** |
| --- | --- |
| Western Regional Hospital | 9,427 |
| Mid-Western Regional Hospital | 3,139 |
| Bardiya District Hospital | 1,065 |
| Bharatpur Hospital | 11,318 |
| Seti Zonal Hospital | 5,767 |
| Nuwakot District Hospital | 1,438 |
| Koshi Zonal Hospital | 8,355 |
| Rapti Sub-Regional Hospital | 3,280 |
| Nawalparasi District Hospital | 1,374 |
| Lumbini Zonal Hospital | 9,007 |
| Bheri Zonal Hospital | 4,276 |
| Pyuthan District Hospital | 1,194 |
